# Gut hormones in POTS and their relation to hemodynamic parameters and gastrointestinal symptoms

**DOI:** 10.1038/s41598-026-52963-0

**Published:** 2026-05-19

**Authors:** Hanna Tufvesson, Bodil Roth, Madeleine Johansson, Viktor Hamrefors, Artur Fedorowski, Eero Lindholm, Bodil Ohlsson

**Affiliations:** 1 Department of Clinical Sciences, Lund University, Lund, Sweden; 2 Department of Gastroenterology, Skåne University Hospital, Malmö, Sweden; 3 Department of Cardiology, Skåne University Hospital, Malmö, Sweden; 4 Department of Medicine, Karolinska Institute, Stockholm, Sweden; 5 Department of Endocrinology, Skåne University Hospital, Malmö, Sweden; 6 Department of Internal Medicine, Skåne University Hospital, Malmö, Sweden

**Keywords:** Postural orthostatic tachycardia (POTS), Glycated hemoglobin, Insulin, Gastrointestinal, Blood pressure, Cortisol, Biomarkers, Diseases, Endocrinology, Gastroenterology, Medical research, Physiology

## Abstract

Postural orthostatic tachycardia syndrome (POTS) is associated with multiple autonomic symptoms, including gastrointestinal (GI) complaints, and has been linked to insulin resistance. We aimed to explore HbA1c, circulating metabolic hormones (insulin, C-peptide, GIP, GLP-1, glucagon, leptin, and peptide YY) and cortisol, and their associations with hemodynamic parameters and GI symptoms in POTS. Two POTS cohorts were studied and compared with matched controls. In the fasting cohort, blood samples were drawn in 42 patients and 41 controls, followed by active standing tests with measurement of pulse and blood pressure (BP) in supine and standing positions. In the non-fasting cohort, questionnaires assessed GI symptoms and HbA1c was measured in 43 patients and 52 controls. Fasting C-peptide and insulin levels correlated with BP in POTS (q = 0.002) but not in controls. Fasting insulin tended to be higher in POTS but was not statistically significant after adjustment for BMI (β = 6.85; 95% CI: −1.04–14.74; *p* = 0.085). Morning cortisol was comparable between groups. In the non-fasting cohort, HbA1c and metabolic hormones were comparable between groups, with no associations with GI symptoms. Together, these findings suggest a potential link between insulin-related pathways and BP regulation in POTS. Future studies are warranted to further investigate insulin dynamics in POTS.

**Supplementary Information:**

The online version contains supplementary material available at 10.1038/s41598-026-52963-0.

## Introduction

Postural orthostatic tachycardia syndrome (POTS) is a common autonomic disorder of the cardiovascular system characterized by abnormal heart rate (HR) increase upon standing, without significant drop in blood pressure (BP)^1^. The definition and characteristics of the syndrome have been described previously^1,2^. The underlying etiology of POTS is not fully understood; however, increased central sympathetic activity and peripheral small fiber neuropathy are proposed mechanisms^2^. Disturbances of glucose homeostasis and insulin resistance have been described as contributing factors in the development of neuropathy, even in the absence of diabetes^3^. Recently, insulin resistance was found in a small POTS cohort following oral glucose tolerance test^4^. The same study showed increased levels of glucose-dependent insulinotropic polypeptide (GIP), pancreatic polypeptide, and peptide YY (PYY) in POTS after oral glucose tolerance test, and there was a time-dependent correlation between levels of GIP and decreased stroke volume/increased HR after tilt test. These findings indicate that metabolic hormones affect splanchnic and/or systemic circulation and may have a pathogenetic role in POTS^4^. Increased blood flow in the pelvic and splanchnic circulation after orthostatic challenge has been demonstrated in POTS in previous studies^5^. The levels of Hemoglobin A1c (HbA1c), which is a marker of long-term glycemic control^6^, have, to our knowledge, never been examined in POTS.

Gastrointestinal (GI) symptoms are common in POTS, where 79% report nausea and 70% report abdominal fullness^7,8^. Disturbances of gastric emptying and antroduodenal manometric abnormalities have been described, suggesting involvement of the enteric nervous system^9,10^. Metabolic hormones, such as GLP-1, may also inhibit gastric emptyping, which may contribute to nausea and fullness^11^. One of the most bothersome GI symptoms in POTS is constipation^8^. Previous studies have shown increased colonic production of PYY in patients with slow transit constipation, although the circulating levels were unaffected^12^. Decreased GLP-1 levels were associated with abdominal pain in patients with constipation-predominant irritable bowel syndrome (IBS)^13^. IBS is a common comorbid disease in POTS^14^. Previous studies have shown impaired glucose tolerance^15^, elevated C-peptide levels in combination with slightly elevated cortisol levels^16^, and alterations of entero-endocrine cells throughout the gut in IBS^17^. Importantly, hormones regulating glucose homeostasis^11^, nutrient intake^18,19^, splanchnic circulation^4^, and motility^20^ are produced in the gut^11^. It is not known whether GI symptoms in POTS are associated with abnormal circulating levels of metabolic hormones.

Given this background information, we hypothesized that long-term disturbances of glucose homeostasis can be found in POTS. In addition, we hypothesized that GI symptoms and hemodynamic abnormalities, which are possible surrogate markers of autonomic neuropathy in POTS, are associated with disturbances of glucose homeostasis and hormones regulating GI function and glucose homeostasis. Accordingly, the aim of the present study was to investigate HbA1c as well as fasting and non-fasting circulating levels of hormones that regulate glucose homeostasis and GI function in POTS and healthy controls.

## Materials and methods

### Study design and study population

**Fig. 1:**
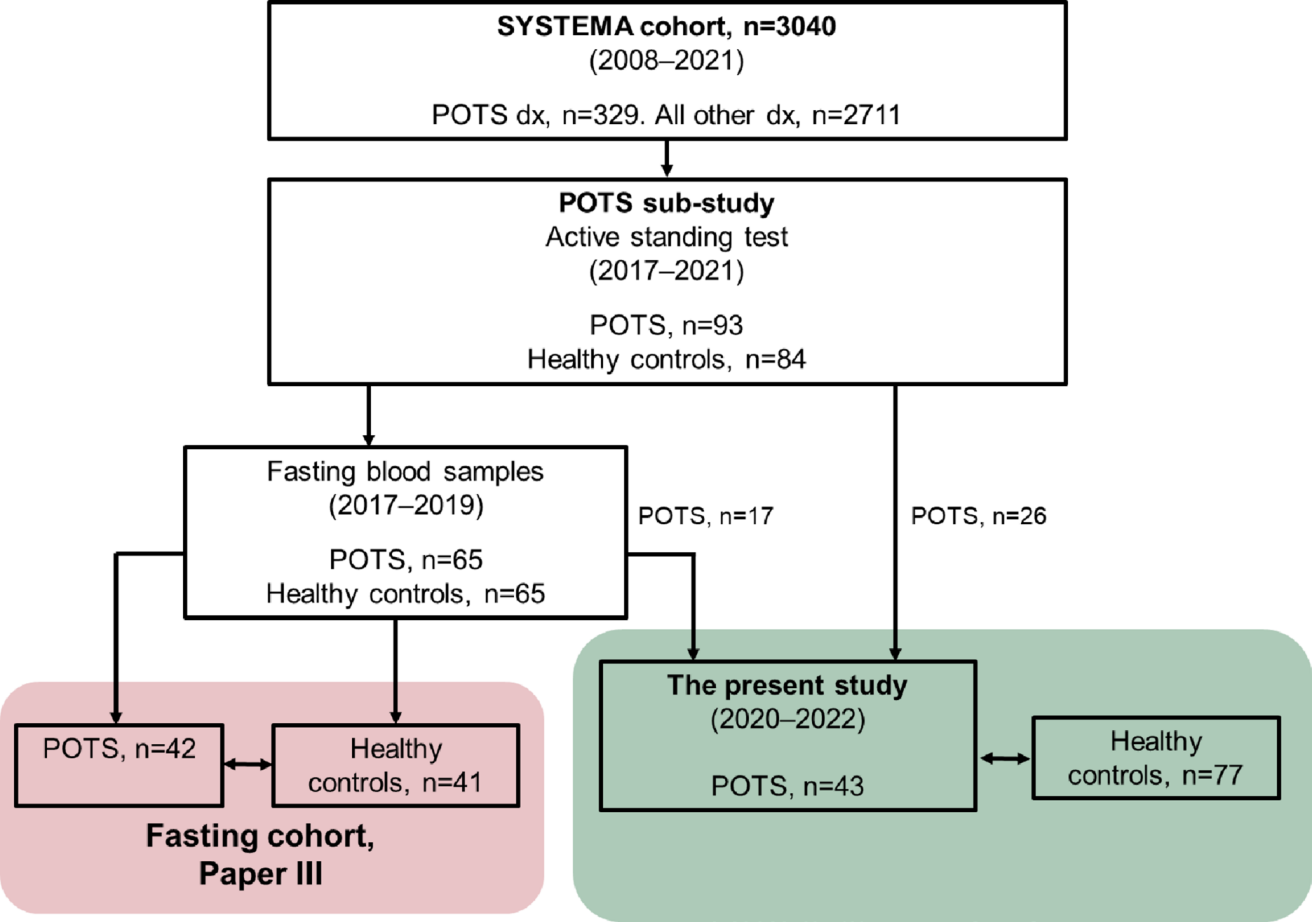
Flowchart on study population recruitment. SYSTEMA: Syncope study of unselected population in Malmö. POTS dx: postural orthostatic tachycardia syndrome diagnosis. GI: gastrointestinal.

The participants were originally recruited from the Syncope Study of Unselected Population in Malmö (SYSTEMA) cohort, with patients referred for unexplained syncope or severe orthostatic intolerance^21^. POTS diagnosis was defined as symptoms of orthostatic intolerance lasting for ≥ 3 months associated with a pathological tilt test showing a HR increase > 30 bpm from supine position or a HR > 120 bpm upon standing, with no evidence of orthostatic hypotension^22^. Patients with a clinically confirmed POTS diagnosis (*n* = 93) from the SYSTEMA cohort, and healthy controls (*n* = 84) were included in the following POTS sub-study, as described earlier^23,24^, at the Clinical Research Unit, Skåne University Hospital, Malmö, Sweden, during 2017–2021. In the present cross-sectional study, two partially overlapping cohorts from the POTS sub-study were included for examination and blood sampling to explore levels of HbA1c and gut hormones in POTS; one fasting cohort^23^, and one non-fasting cohort^8^(Fig. 1). Fasting blood samples were obtained between 2017 and 2019 at the previous examination within the POTS sub-study. The participants underwent active standing test immediately after blood sampling^25^. Levels of fasting metabolic hormones were compared between patients and healthy controls, and correlations were examined between hormonal levels and hemodynamic findings derived from the active standing tests. Non-fasting blood samples were obtained for this specific study 2020–2022 while patients recruited from the POTS sub-study were re-examined and filled out GI questionnaires at the Department of Gastroenterology, Skåne University Hospital, Malmö. Healthy control participants were recruited separately for this cohort (Fig. 1). Levels of HbA1c and metabolic hormones were compared between patients and healthy controls, and correlations were examined between laboratory parameters and results from questionnaires.

### Fasting cohort

All patients had a clinically defined diagnosis of POTS according to international consensus^2,22^. Exclusion criteria were other primary causes of the hemodynamic findings. Healthy controls without a history of syncope, orthostatic intolerance, cancer, cardiovascular or endocrine diseases were recruited among volunteers, students, and hospital staff members. The participants were instructed to fast overnight, avoid smoking, and discontinue all cardiovascular pharmacological agents such as beta-blockers, ivabradine, midodrine and droxidopa 48 h prior to examination. Blood samples were drawn with the participant in supine position in the morning between 8 AM and 11 AM preceding the active standing test. The controls did not have any significant HR increasement during active standing test^23^. In total, fasting blood samples were obtained in 65 patients with POTS and 65 controls. To the present study, fasting serum and plasma stored and frozen in aliquots at −80 °C were collected from 42 randomly selected POTS patients and 41 age- and sex-matched healthy controls, and these participants constituted the fasting cohort (Fig. 1). The frozen aliquots (225 µL) had never been thawed before.

### Non-fasting cohort

Patients with a definite POTS diagnosis, as described in the previous sections, age 18–70 years, were invited to the re-examination study of POTS. Exclusion criteria were severe psychiatric or somatic comorbidity, inability to understand study information, and severe opioid or alcohol abuse. Healthy control participants with no chronic diseases or pharmacological treatment were recruited separately to this study among students, hospital staff, and their relatives (Fig. 1). Details of the recruitment process have been described previously^8^. The patients took their regular medications prior to the study visit. Seventeen of the patients who were included in this study cohort were also participants of the cohort where fasting blood samples were drawn between 2017 and 2019 (Fig. 1).

A physician (HT) performed a clinical examination of POTS patients during the study visit, including heart-, lung-, abdominal-, and neurological status, current weight, height, as well as resting BP from the right arm, in a supine position after approximately 5 min of rest. Healthy controls were examined with height and weight. All participants filled out questionnaires, and blood samples were drawn with the participants in supine positions. All examinations and blood sampling of POTS patients and controls were conducted approximately during the same time span of the day, between 9 am and 3 pm, and recent food intake status was not recorded. Blood samples were collected in ethylenediaminetetraacetic acid (EDTA) tubes (BD Microtainer, Franklin Lakes, NJ, USA), cooled, and centrifuged at 3,000 relative centrifugal force for 5 min. The plasma was harvested and frozen in 225 µL aliquots at −80 °C after collection.

##  Questionnaires

###  Study questionnaire

The participants from the non-fasting cohort were asked to complete a study questionnaire regarding sociodemographic factors, lifestyle habits, previous and current illnesses, family history, and current pharmacological treatment, as well as GI symptom evaluation as described below.

### The visual analog scale for irritable bowel syndrome (VAS-IBS)

The VAS-IBS is a validated questionnaire regarding GI symptoms common in IBS^26^. The respondent marks their degree of symptoms for the past 2 weeks on a visual analog scale (VAS) between 0 and 100 mm, where 100 mm means very severe symptoms. The items covered are abdominal pain, diarrhea, constipation, bloating and flatulence, vomiting and nausea, and the intestinal symptoms’ influence on daily life. The degree of psychological well-being is also included in the questionnaire. The scales are inverted from the original version^26^.

### The irritable bowel syndrome severity scoring system (IBS-SSS)

The IBS-SSS questionnaire is a validated scoring system used in IBS, consisting of four items regarding abdominal pain, abdominal distension, satisfaction with bowel habit, and the impact of bowel habits on daily life, measured in mm on a 0–100 mm VAS, where 100 mm means very severe symptoms. In addition, one question asks the number of days of abdominal pain in the past 10 days. Scores from these questions are added, producing total IBS-SSS, with a maximum score of 500. Scores between 75 and 175 suggests mild IBS, 175–300 suggest moderate IBS, and > 300 suggest severe IBS^27^.

## Active standing test

All participants from the POTS sub-study were examined at the Clinical Research Unit between 2017 and 2021 (Fig. 1), where they performed an active standing test, with a 10-minute rest in the supine position prior to standing. BP and HR were measured twice in the supine position by a validated automated oscillometer device (OMRON Healthcare Co., Ltd., Kyoto, Japan), and then after 1, 3, and 5 min of standing. An average of two measurements in the supine position was used for group comparisons. For orthostatic HR increase, difference between standing HR after 1, 3, and 5 min, and supine HR was calculated, producing ΔHR_1min_, ΔHR_3min_, and ΔHR_5min_, respectively. The hemodynamic parameters used when examining possible correlations to hormonal levels were supine systolic and diastolic BP (SBP and DBP), supine HR, and SBP, DBP, and ΔHR at 1, 3, and 5 min of standing.

## Laboratory analyses

### Basic biochemical tests

HbA1c (mmol/mol) was analyzed from the non-fasting cohort, according to clinical routines at the Department of Laboratory Medicine, Skåne University Hospital, Malmö, Sweden^28^. Hemoglobin levels (Hb, g/L) and erythrocyte mean corpuscular volume (erc-MCV, fL) were analyzed in blood from the non-fasting cohort according to clinical routines^29^, to exclude underlying hemoglobinopathies that can affect levels of HbA1c^6^. Morning plasma cortisol (nmol/L) was measured in fasting plasma samples according to clinical routines at the Department of Laboratory medicine, Skåne University Hospital^30^.

###  Gut hormonal analyses

The Mesoscale Discovery (MSD, Rockville, MD, USA) U-PLEX Human Diabetes Combo 1 multiplex assay (K15274K) was used to perform analyses of C-peptide, GIP (total), glucagon-like peptide-1 (GLP-1,total), glucagon, insulin, leptin and PYY (total) in serum (fasting blood samples) and EDTA plasma (non-fasting blood samples) by electro-chemiluminescence detection. For each hormone a biotinylated capture antibody was coupled to a specific linker, 50 µL antibody solution/well were added to U-PLEX multiplex plate and incubated overnight at 4℃ on a shaker. A metabolic assay working solution containing aprotinin, DPP-IV inhibitor (Merck, Darmstadt, Germany), and diluent was prepared for dilution of the calibrator and serum or EDTA plasma sample. Calibrators (C-peptide 5.57–282,000 pg/mL, GIP 30.8–126,000 pg/mL, GLP-1 1.79–7350 pM, glucagon 0.38–1,570 pM, insulin 1.79–7,340 µU/mL, leptin 119–488,000 pg/mL and PYY 5.57–22,800 pg/mL), 50 µL/well, and serum or EDTA plasma (diluted 1:2) were added after the plates had been washed three times with MSD wash buffer. A one-hour incubation in room temperature on a shaker was followed by a new washing procedure and a SULFO-TAG detection antibody, 50 µL/well, was added. After a new one-hour incubation and a washing procedure, 150 µL MSD GOLD Read Buffer B in each well was added and the plates were read on an MSD instrument.

The intensity of emitted light is proportional to the amount of C-peptide, GIP, GLP-1, glucagon, insulin, leptin and PYY in the wells.

Four of the samples were analyzed in both EDTA plasma and serum with good agreement on all hormones except GIP, where serum levels were significantly lower in serum compared to EDTA plasma (Supplementary Table S1). The intra- and inter-assay coefficients of variance (CV) are shown in Supplementary Table S2. The CV-values have been calculated by The Mesoscale Discovery based on six previous samples, and at three different dilutions, and not on samples from the current study.

## Statistical analyses

Statistical analyses were performed using IBM Statistical Package for the Social Sciences (SPSS) Statistics (version 29.0, IBM, Armonk, NY, USA), available at: https://www.ibm.com/products/spss-statistics. The Shapiro-Wilk test was used to check data for normality. Most variables were non-normally distributed within POTS and healthy controls in the fasting and non-fasting cohorts. Therefore, Mann-Whitney U test and Fisher’s exact test or Chi2-test were used for comparisons between groups, as appropriate. Multiple linear regression was used to adjust for confounders such as sex, age, and body mass index (BMI). Spearman’s correlation test was used to assess correlations. Differences in correlation coefficients between groups were assessed using Fisher’s r-to-z transformation. Although this method is formally derived for Pearson correlations, it was applied to Spearman correlation coefficients as a practical solution. To adjust for multiple comparisons in the correlation analyses, crude p-values < 0.05 were adjusted for false discovery rate (FDR) set at α = 0.05 according to the Benjamini-Hochberg procedure^31^. The FDR-adjusted p-values, indicated as q, were the main results. A sensitivity analysis was performed in the fasting cohort with exclusion of individuals who reported use of medicines that likely had the potential to interfere with glucose homeostasis, or use of medicines that could indicate metabolic syndrome disorders^32–36^. These included betablocker use < 48 h before blood sampling, use of tricyclic antidepressants (TCA), oral combined contraceptives, oral budesonide, anti-diabetic medicines, and statins. Values are presented as median and interquartile range (IQR) or numbers and percentages, or β and 95% confidence interval (CI). P or q < 0.05 was considered statistically significant.

## Ethics

The present study was performed in accordance with the Declaration of Helsinki. The Regional Ethical Committee in Lund, Sweden, approved the SYSTEMA project (82/2008) and the POTS sub-study (2017/295). The re-examination study that included clinical examinations, non-fasting blood samples, and questionnaires was approved by the Swedish Ethical Review Authority, Dnr 2020–02432 and 2021–00049. Written and informed consent was provided by all subjects before participation in the studies.

## Results

### Fasting analyses

Forty-two patients with POTS and 41 healthy controls matched for age and gender were included in the study, where blood samples were drawn on the occasion for active standing test. The median BMI was equal in the two groups, however, data on current BMI was only reported in 19 of the 42 POTS patients, and in eight of the control participants (Table 1). Patients were asked not to take any cardiovascular agents 48 h before the examination; however, twelve (29%) patients were unable to abstain from medication due to pronounced symptoms. Medications are shown in Supplementary Table S3. Selective and/or non-selective beta-blocker use was reported by 17 (40%) patients. Of these, seven (17%) were taking the medicines < 48 h before examination. Three patients reported use of TCA, four reported combined oral contraceptives, one reported metformin, one reported oral budesonide, and one reported use of statins. Use of peripheral and/or central acting sympathomimetic medicines was reported by 19 (45%) POTS patients. Of these, two (5%) were taking the medicines < 48 h before examination.

**Table 1:** Clinical characteristics, levels of serum metabolic hormones and morning plasma cortisol in fasting POTS patients and healthy controls.

	POTS, *n* = 42	Healthy controls, *n* = 41	*p*-value
Age (years)	26 (23–37)	28 (24–37)	0.548
BMI (kg/m ^2^ )	23.5 (20.5–28.7)^a^	23.2 (21.0–26.1)^b^	0.595
Female, n (%)	38 (90.5%)	36 (87.8%)	0.738
C-peptide (pg/mL)	1,642 (1,148–2,202)	1,315 (1,084–1,677)^c^	0.054
GIP total (pg/mL)	37.8 (25.5–65.0)	32.3 (23.8–62.2)^d^	0.257
GLP-1 (pM)	9.5 (78–11.0)	8.6 (7.1–10.3)^c^	0.113
Glucagon (pM)	14.9 (10.3–18.8)	12.7 (10.0–16.5)^c^	0.202
Insulin (µU/mL)	14.8 (11.0–19.2)	11.9 (9.1–15.7)^c^	0.029
Leptin (pg/mL)	22,831 (12,591–42,052)	19,922 (10,798–31,825)^c^	0.342
PYY (pg/mL)	49.5 (35.0–75.1)	47.4 (33.3–57.3)^c^	0.358
Cortisol (nL)	424 (278–528)	404 (298–570)	0.753

Values presented as number (percent) or median (interquartile range). Comparative analyses were performed with Fisher’s Exact test or Mann Whitney-U test. A p-value < 0.05 was considered statistically significant. GI: gastrointestinal; POTS: postural orthostatic tachycardia syndrome; BMI: Body mass index; GIP: glucose-dependent insulinotropic polypeptide; GLP-1: glucagon-like peptide-1; PYY: peptide YY.

^a^23 missing values.

^b^33 missing values.

^c^1 missing value.

^d^2 missing values.

Hemodynamic data from the active standing tests are presented in Table 2. The median HR was higher in POTS at all measure points compared with controls. ΔHR was higher in POTS compared with controls at all measure points, however, the significance level was not reached after 5 min of standing. DBP was higher in POTS compared with controls after 1 and 3 min of standing.

**Table 2:** Data on active standing in the fasting cohort of POTS patients and healthy controls.

	POTS, *n* = 42	Healthy controls, *n* = 41	*P*-value
SBP supine	112 (107–120)^a^	112 (107–118)	0.587
DBP supine	68 (64–75)^a^	69 (63–73)	0.341
HR supine	70 (63–83)^a^	61 (56–69)	0.002
SBP 1 min	116 (108–125)^b^	114 (104–118)	0.111
DBP 1 min	81 (75–86)^b^	76 (68–82)	0.036
HR 1 min	93 (80–108)^b^	80 (68–91)	< 0.001
ΔHR _1 min_	20 (14–33)^b^	16 (7–27)	0.017
SBP 3 min	116 (107–124)	114 (105–120)	0.159
DBP 3 min	80 (71–87)	77 (68–80)	0.039
HR 3 min	93 (84–107)	84 (71–97)	< 0.001
ΔHR _3 min_	26 (17–32)	21 (10–27)	0.014
SBP 5 min	111 (106–122)^c^	110 (102–120)^a^	0.195
DBP 5 min	77 (70–85)^c^	73 (66–81)^a^	0.056
HR 5 min	94 (83–111)^c^	86 (73–94)^a^	0.003
ΔHR _5 min_	25 (18–34)^c^	21 (15–30)^a^	0.101

Values presented as median (interquartile range). Comparative analyses were performed with Mann Whitney-U test. A p-value < 0.05 was considered statistically significant. Blood pressure measured in mmHg and heart rate measured in beats per minute. POTS: postural orthostatic tachycardia syndrome; SBP: systolic blood pressure; DBP: diastolic blood pressure; HR: heart rate.

^a^1 missing value.

^b^4 missing values.

^c^5 missing values.

The level of serum insulin was significantly higher in POTS compared with controls (14.8 [11.0–19.2] vs. 11.9 [9.1–15.7] µU/mL; *p* = 0.029) and slightly elevated according to a large previous study on healthy women, where reference interval was set at 2.54–13.30 µU/mL^37^. After adjustment for sex and age, insulin was still higher in POTS than controls, β = 3.55; 95% CI: 0.39–6.72; *p* = 0.028. However, when adding adjustment for BMI, this difference disappeared, β = 6.85; 95% CI: −1.04–14.74; *p* = 0.085. C-peptide was also higher in POTS, although the significance level was not reached (*p* = 0.054, Table 1). After adjustment for sex and age, C-peptide was higher in POTS than in controls, β = 298; 95% CI: 42–553; *p* = 0.023. However, when adding adjustment for BMI, this difference disappeared, β = 495; 95% CI: −55.6–1046; *p* = 0.076. Otherwise, there were no significant differences between POTS and controls regarding metabolic hormones (Table 1).

**Fig. 2:**
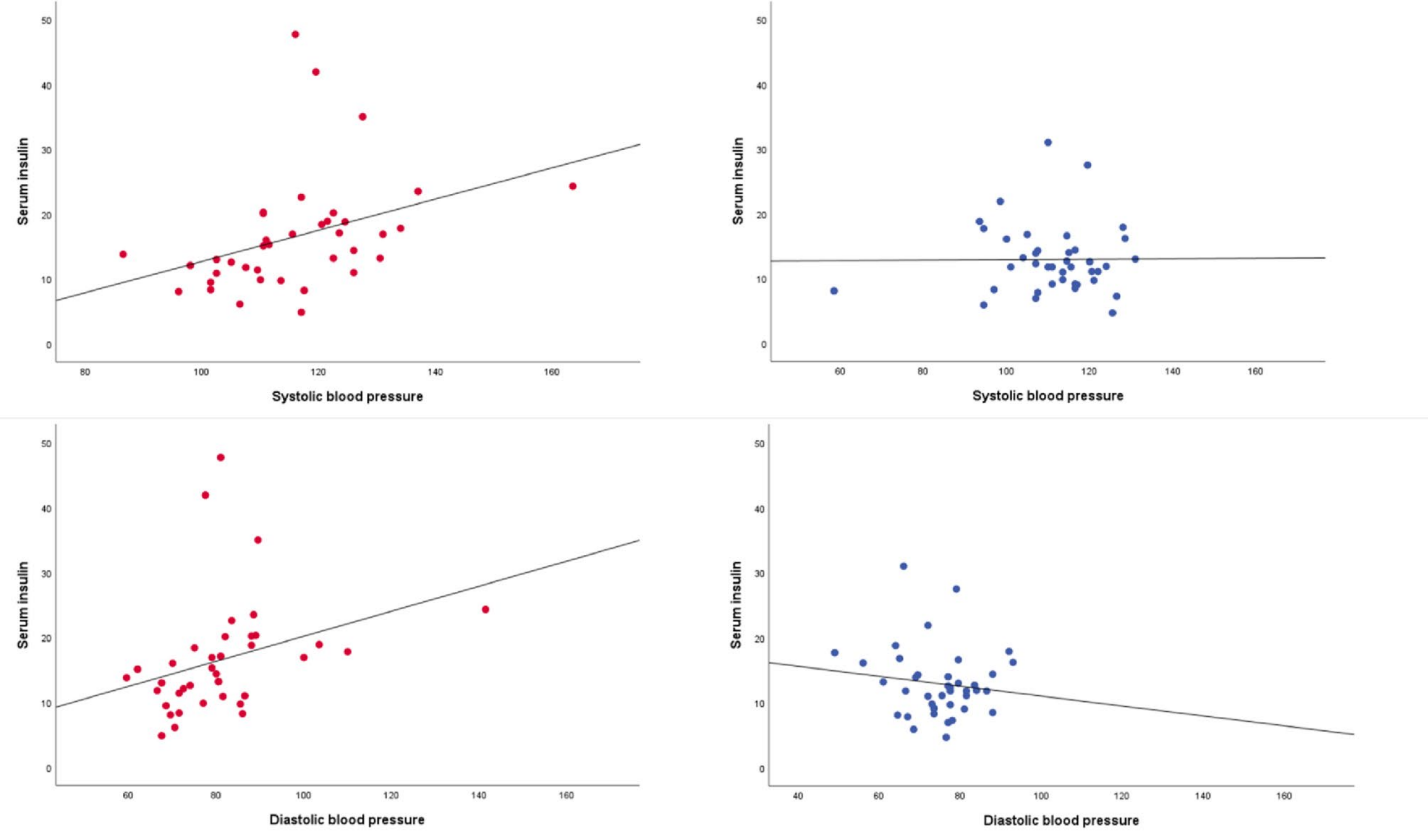
Correlations between serum insulin and systolic/diastolic blood pressure in POTS and healthy controls. Scatterplots showing the correlations (also shown in Table 3) between fasting insulin and systolic/diastolic blood pressure in patients with POTS (left panel) and controls (right panel). Lines represent linear regression lines, used for illustration. Serum insulin (µU/mL) on the y-axis. Systolic/diastolic blood pressure (mmHg) after 3 min of standing on the x-axis.

According to Spearman’s Rho, there were moderate positive correlations between levels of C-peptide/insulin, and SBP/DBP at all measure points in POTS, after adjusting p-values with FDR (Fig. 2; Table 3). The correlations between fasting C-peptide/insulin and BP measures were significantly stronger in patients with POTS compared with controls at almost all measure points (Table 3). There were significant positive correlations between levels of leptin and SBP/DBP at some of the measure points in POTS and controls, but this pattern did not differ between groups (Table 3). There were no significant correlations between insulin, C-peptide, or leptin and HR or ΔHR (Table 3).

**Table 3:** Correlations between fasting metabolic hormonal levels and hemodynamic parameters during active standing test in POTS and controls.

	C-peptide			Insulin			Leptin		
	POTS *N* = 42	Controls *N* = 40	Fisher *r* to z	POTS	Controls	Fisher *r* to z	POTS	Controls	Fisher *r* to z
**SBP**_**sup**_	*r* = 0.516^a^ *p* < 0.001 q = 0.002	*r* = 0.192^a^ *p* = 0.235 q = 0.554	z = 1.994 *p* = 0.046 q = 0.053	*r* = 0.496^a^ *p* < 0.001 q = 0.002	*r* = 0.110^a^ *p* = 0.500 q = 0.863	z = 1.877 *p* = 0.061 q = 0.070	*r* = 0.423^a^ *p* = 0.006 q = 0.038	*r* = 0.164^a^ *p* = 0.313 q = 0.360	z = 1.238 *p* = 0.216
**DBP**_**sup**_	*r* = 0.562^a^ *p* < 0.001 q = 0.002	*r* = 0.256^a^ *p* = 0.110 q = 0.554	z = 1.619 *p* = 0.105 q = 0.105	*r* = 0.533^a^ *p* < 0.001 q = 0.002	*r* = 0.261^a^ *p* = 0.104 q = 0.572	z = 1.416 *p* = 0.157 q = 0.157	*r* = 0.375^a^ *p* = 0.016 q = 0.038	*r* = 0.334^a^ *p* = 0.035 q = 0.086	z = 1.083 *p* = 0.855
**HR**_**sup**_	*r* = 0.216^a^ *p* = 0.175 q = 0.233	*r* = 0.178^a^ *p* = 0.272 q = 0.554		*r* = 0.273^a^ *p* = 0.084 q = 0.112	*r* = 0.186^a^ *p* = 0.250 q = 0.706		*r* = 0.119^a^ *p* = 0.459 q = 0.551	*r* = 0.333^a^ *p* = 0.036 q = 0.086	
**SBP**_**1**_	*r* = 0.595^b^ *p* < 0.001 q = 0.002	*r* = 0.086^a^ *p* = 0.599 q = 0.719	z = 2.541 *p* = 0.011 q = 0.032	*r* = 0.597^b^ *p* < 0.001 q = 0.002	*r*=−0.006^a^ *p* = 0.972 q = 0.972	z = 2.954 *p* = 0.003 q = 0.006	*r* = 0.331^b^ *p* = 0.042 q = 0.072	*r* = 0.192^a^ *p* = 0.236 q = 0.360	z = 0.634 *p* = 0.526
**DBP**_**1**_	*r* = 0.599^b^ *p* < 0.001 q = 0.002	*r* = 0.160^a^ *p* = 0.323 q = 0.554	z = 2.249 *p* = 0.025 q = 0.040	*r* = 0.574^b^ *p* < 0.001 q = 0.002	*r* = 0.035^a^ *p* = 0.828 q = 0.903	z = 2.623 *p* = 0.009 q = 0.012	*r* = 0.412^b^ *p* = 0.010 q = 0.038	*r* = 0.404^a^ *p* = 0.010 q = 0.040	z = 0.041 *p* = 0.968
**ΔHR**_**1**_	*r*=−0.110^b^ *p* = 0.511 q = 0.613	*r* = 0.169^a^ *p* = 0.298 q = 0.554		*r*=−0.073^b^ *p* = 0.663 q = 0.723	*r* = 0.170^a^ *p* = 0.294 q = 0.706		*r*=−0.178^b^ *p* = 0.286 q = 0.381	*r*=−0.163^a^ *p* = 0.314 q = 0.360	
**SBP**_**3**_	*r* = 0.561^b^ *p* < 0.001 q = 0.002	*r* = 0.123^a^ *p* = 0.451 q = 0.651	z = 2.166 *p* = 0.030 q = 0.040	*r* = 0.547^b^ *p* < 0.001 q = 0.002	*r*=−0.075^a^ *p* = 0.647 q = 0.863	z = 2.923 *p* = 0.003 q = 0.006	*r* = 0.356^b^ *p* = 0.028 q = 0.056	*r* = 0.181^a^ *p* = 0.263 q = 0.360	z = 0.803 *p* = 0.422
**DBP**_**3**_	*r* = 0.609^b^ *p* < 0.001 q = 0.002	*r* = 0.113^a^ *p* = 0.488 q = 0.651	z = 2.519 *p* = 0.012 q = 0.032	*r* = 0.556^b^ *p* < 0.001 q = 0.002	*r*=−0.057^a^ *p* = 0.725 q = 0.870	z = 2.901 *p* = 0.004 q = 0.006	*r* = 0.390^b^ *p* = 0.016 q = 0.038	*r* = 0.440^a^ *p* = 0.005 q = 0.040	z=−0.257 *p* = 0.798
**ΔHR**_**3**_	*r*=−0.066^b^ *p* = 0.695 q = 0.758	*r* = 0.238^a^ *p* = 0.139 q = 0.554		*r*=−0.025^b^ *p* = 0.883 q = 0.883	*r*=−0.236^a^ *p* = 0.143 q = 0.572		*r* = 0.054^b^ *p* = 0.747 q = 0.815	*r*=−0.158^a^ *p* = 0.330 q = 0.360	
**SBP**_**5**_	*r* = 0.627^c^ *p* < 0.001 q = 0.002	*r* = 0.023^d^ *p* = 0.888 q = 0.969	z = 2.960 *p* = 0.003 q = 0.024	*r* = 0.627^c^ *p* < 0.001 q = 0.002	*r*=−0.092^d^ *p* = 0.577 q = 0.863	z = 3.439 *p* < 0.001 q = 0.006	*r* = 0.421^c^ *p* = 0.010 q = 0.038	*r* = 0.228^d^ *p* = 0.164 q = 0.328	z = 0.900 *p* = 0.368
**DBP**_**5**_	*r* = 0.504^c^ *p* = 0.001 q = 0.002	*r*=−0.001^d^ *p* = 0.995 q = 0.995	z = 2.306 *p* = 0.021 q = 0.040	*r* = 0.551^c^ *p* < 0.001 q = 0.002	*r*=−0.107^d^ *p* = 0.517 q = 0.863	z = 3.018 *p* = 0.003 q = 0.006	*r* = 0.319^c^ *p* = 0.054 q = 0.081	*r* = 0.408^d^ *p* = 0.010 q = 0.040	z=−0.426 *p* = 0.670
**ΔHR**_**5**_	*r* = 0.046^c^ *p* = 0.787 q = 0.787	*r* = 0.365^d^ *p* = 0.022 q = 0.264		*r*=−0.092^c^ *p* = 0.518 q = 0.622	*r* = 0.414^d^ *p* = 0.009 q = 0.108		*r* = 0.001^c^ *p* = 0.996 q = 0.996	*r* = 0.018^d^ *p* = 0.915 q = 0.915	

Correlations performed with Spearman’s test in 42 patients with POTS and 41 controls. Differences in correlations between groups were calculated with Fisher’s r-to-z test. Q = P-values adjusted for false discovery rate according to the Benjamini-Hochberg procedure^31^. A q < 0.05 was considered significant. POTS: postural orthostatic tachycardia syndrome; SBP: systolic blood pressure; DBP: diastolic blood pressure; HR: heart rate.

^a^1 missing value.

^b^4 missing values.

^c^5 missing values.

^d^2 missing values.

After sensitivity analysis 27 patients remained in the POTS group. The trend with higher insulin in POTS remained, but the results were no longer statistically significant (15.3 [10.0–18.4] vs. 11.9 [9.1–15.7] µU/mL; *p* = 0.061). The same pattern was seen with C-peptide (Supplementary table S4). The correlations between C-peptide/insulin and BP became stronger in POTS after the sensitivity analysis, and the difference remained significant between POTS and controls after Fisher’s r-to-z analysis in almost all measure points regarding insulin, and in some regarding C-peptide (Supplementary Table S5).

There were no correlations between the analyzed hemodynamic parameters and levels of GLP-1, GIP, glucagon or PYY in POTS (Supplementary Table S6).

There was no statistically significant difference in morning plasma cortisol levels between POTS and controls (424 [278–528] vs. 404 [298–570] nmol/L; *p* = 0.753, Table 1) or any correlations between cortisol levels and C-peptide/insulin or SBP/DBP at any of the measure points (Supplementary Table S7).

### Non-fasting analyses

Forty-three patients with POTS and 52 healthy controls were included in the study. The groups were comparable regarding age, gender, and BMI. In this cohort, almost all POTS patients had undergone an active standing test at a previous occasion, but none of the healthy controls. Smoking was equally common in POTS and controls. The controls reported more weekly physical activity than the patients with POTS (*p* = 0.032), (Table 4). Self-reported comorbidities are shown in Supplementary Table S8. The most common self-reported comorbidities in the POTS group were hypermobile spectrum disorders/Ehlers-Danlos syndrome (*n* = 12, 28%), IBS (*n* = 12, 28%), asthma (*n* = 8, 19%), migraine (*n* = 7, 16%), and neuropsychiatric disorders (*n* = 6, 14%). One patient reported type 2 diabetes mellitus, but no anti-diabetic medication. Pharmacological treatment was abundant in the POTS group, especially cardiovascular and anti-allergy drugs. One patient reported using GLP-1-analog, prescribed off-label due to over-weight. Self-reported pharmacological medicines are shown in Supplementary Table S9. GI symptoms and low psychological well-being, measured on the VAS-IBS scale and the IBS-SSS scale, were significantly more severe in POTS compared with controls (Table 5), and this data has also been published recently^8^.

**Table 4:** Clinical characteristics of non-fasting POTS patients and healthy controls.

		POTS, *n* = 43	Healthy controls, *n* = 52	*p*-value
Age (years)		30.6 (26.0–41.0)	34.5 (29.0–39.4)	0.121
Female, n (%)		40 (93.0%)	43 (82.7%)	0.214
BMI (kg/m ^2^ )		24.2 (21.3–27.0)	22.4 (21.0–24.1)	0.161
Smoking, n (%)		^a^	^b^	0.317
	Regularly	5 (11.6%)	1 (2.5%)	
	Sometimes	1 (2.3%)	1 (2.5%)	
	Quit smoking	5 (11.6%)	8 (20.0%)	
	Never smoked	32 (74.4%)	31 (77.5%)	
Weekly physical activity, n (%)			^b^	0.032
	No time at all	8 (18.6%)	2 (5.0%)	
	< 30 min	6 (14.0%)	4 (10.0%)	
	30–60 min	10 (23.3%)	8 (20.0%)	
	60–90 min	11 (25.6%)	5 (12.5%)	
	90–120 min	2 (4.7%)	5 (12.5%)	
	> 120 min	6 (14.0%)	16 (40.0%)	

Values presented as number (percent) or median (interquartile range). Comparative analyses were performed with Chi2-test, Fisher’s Exact test or Mann Whitney-U test. A p-value < 0.05 was considered statistically significant. POTS: postural orthostatic tachycardia syndrome; BMI: body mass index.

^a^1 missing answer.

^b^12 missing answers.

**Table 5:** GI symptoms and levels of, HbA1c, Hb, erc-MCV and plasma metabolic hormones in non-fasting POTS and controls.

	POTS, *n* = 43	Healthy controls, *n* = 52	*p*-value
Total IBS-SSS	213 (135–319)^c^	11 (0–45)	< 0.001
VAS-IBS (mm)			
Abdominal pain	30 (16–62)	0 (0–0)	< 0.001
Diarrhea	28 (0–64)	0 (0–0)	< 0.001
Constipation	61 (11–74)	0 (0–13)	< 0.001
Bloating and flatulence	65 (25–88)	0 (0–9)^a^	< 0.001
Vomiting and nausea	44 (23–70)	0 (0–5)	< 0.001
Psychological well-being	50 (28–62)	11 (0–36)^b^	< 0.001
Symptom’s influence on daily life	58 (23–76)	0 (0–6)	< 0.001
C-peptide (pg/mL)	2,576 (1,782–3,444)^b^	2,176 (1,278–3,272)	0.173
GIP, total (pg/mL)	263.9 (107.5–573.1)^b^	245.7 (118.6–625.0)	0.593
GLP-1, total (pM)	17.6 (13.1–30.8)^b^	16.8 (12.4–23.6)	0.528
Glucagon (pM)	18.4 (11.9–24.2)^b^	15.6 (11.2–21.4)	0.200
Insulin (µU/mL)	25.9 (13.9–43.0)^b^	20.4 (11.8–40.3)	0.496
Leptin (pg/mL)	16,645 (8,879–47,871)^b^	11,041 (5,143–20,463)	0.003
PYY, total (pg/mL)	79.3 (50.7–120.7)^b^	77.4 (60.6–101.5)	0.710
HbA1c (mmol/mol)	33.0 (31.0–35.0)^d^	33.5 (32.0–35.0)^e^	0.423
Hb (g/L)	131 (125–140)^c^	132 (122–141)^b^	0.938
Erc-MCV (fL)	90 (88–92)^c^	91 (87–93)^b^	0.471

Values are presented as median (interquartile range). Comparisons were performed with Mann Whitney U-test. *P* < 0.05 was considered statistically significant. GI: gastrointestinal; POTS: postural orthostatic tachycardia syndrome; IBS-SSS: irritable bowel syndrome severity scoring system^27^; VAS-IBS: visual analog scale for irritable bowel syndrome^26^; GIP: glucose-dependent insulinotropic peptide; GLP-1: glucagon-like peptide-1; PYY: peptide YY; HbA1c: hemoglobin A1c; Hb: hemoglobin; Erc-MCV: erythrocyte mean corpuscular volume.

^a^1 missing value.

^b^2 missing values.

^c^3 missing values.

^d^4 missing values.

^e^14 missing values.

There were no significant differences regarding levels of HbA1c, Hb and erc-MCV between POTS and controls. Neither were there any differences in levels of C-peptide, GIP, GLP-1, insulin, glucagon, and PYY between POTS and controls. The leptin level in plasma was significantly higher in POTS compared with controls (16,645 [8,879–47,871] vs. 11,041 [5,143–20,463] pg/mL; *p* = 0.003) (Table 5). However, when adjusting for age, gender and BMI, the association between POTS and leptin disappeared (β = 5,638; 95%CI: −1,395–12,670; *p* = 0.114).

There were no correlations between levels of HbA1c and any of the analyzed gut hormones (C-peptide, insulin, glucagon, GIP, GLP-1, PYY, and leptin) (Supplementary Table S10). No correlations were seen between GI symptoms and BMI in POTS (Supplementary Table S11). Likewise, there were no significant correlations between the non-fasting metabolic hormones and the GI symptoms measured on the VAS-scale and total IBS-SSS in POTS (Supplementary Table S12).

## Discussion

The main finding of the present study was the presence of positive correlations between fasting C-peptide/insulin levels and BP measures in patients with POTS, but not in healthy controls. In contrast, HbA1c levels were similar between groups, suggesting no differences in long-term glycemic control. Although fasting insulin levels appeared higher in POTS, the difference was no longer significant after adjustment for BMI. Furthermore, no associations were found between fasting metabolic hormone levels and HR increase, nor between non-fasting metabolic hormone levels and GI symptoms in POTS.

Elevated insulin levels have been found in POTS in a previous study by Breier et al^4^.. In another small study on endothelial dysfunction in POTS, levels of insulin and glucose were higher in POTS than in healthy controls, but the differences were not significant^38^, in line with the findings of the present study. Insulin resistance and/or signs of pre-diabetes have been linked to comorbidities commonly encountered in POTS^14^, such as migraine^39^, and IBS^15,40^. However, the present study was not primarily designed for testing insulin resistance. Taken together, alterations in insulin dynamics in POTS are subtle, context-dependent, and more apparent in relation to cardiovascular parameters rather than baseline group differences.

Insulin is a potent hormone which is strongly associated with glucose- and lipid metabolism, with widespread effects all over the human body^41,42^. Increased insulin levels are found in several other diverse clinical settings. Probably the most known and common condition associated with hyperinsulinemia and insulin resistance is the metabolic syndrome, characterized by dyslipidemia, visceral adiposity, and hypertension^43^. The pathophysiological mechanisms are complex and include abnormalities in vasoactive agents such as nitric oxide (NO) and endothelin, leptin levels and activation of sympathetic nervous system (SNS), including increased levels of norepinephrine^42–48^. We did not find any evidence of manifest hypertension, and the BMI and leptin levels, as a proxy for adipose tissue mass^49^, were comparable and within normal distribution in POTS and controls. However, the present study was not designed to evaluate all aspects of metabolic syndrome.

Activation of SNS is known to induce insulin production by stimulation of hepatic glucose release and inhibition of peripheral glucose uptake^50^. This mechanism may provide a possible explanation for the observed correlation between fasting insulin levels and BP in POTS in the present study, potentially reflecting a shared link through autonomic dysregulation.

Furthermore, cortisol increases blood glucose and insulin during stress and is also associated with increased BP^51,52^. The adrenal cortex function has been shown to be normal in POTS, with a normal hormonal response following adrenocorticotropin stimulation^53^. Yet, serum and salivary cortisol levels have been increased in previous studies on POTS^54,55^. After analysis of the results from the present study, we decided to add measurement of cortisol from the remaining frozen morning plasma obtained from the fasting POTS cohort and controls. However, we did not find any evidence for increased cortisol levels in POTS and there were neither any correlations between cortisol levels and C-peptide/insulin, nor between cortisol and SBP/DBP. Thus, cortisol is less likely to explain the findings regarding correlation of fasting C-peptide/insulin levels and BP in POTS in the present study.

Some drugs are associated with higher levels of blood glucose and insulin resistance, including treatment with betablockers^34–36^. In addition, treatment with TCA is associated with increased blood glucose, as is treatment with steroids, and combined oral contraceptives^34–36^. It cannot be excluded that the findings regarding insulin dynamics in the present study were mediated by medicine effects or comorbidities in the fasting POTS cohort. However, the correlations between insulin and BP remained at all measure points in POTS, and differed significantly from the controls after sensitivity analysis, which strengthens the theories of a biological signal. Theoretically, treatment with sympathomimetic drugs may increase insulin in the same fashion as increased sympathetic tone, but most patients managed to abstain from these medicines at the time of the tests.

To our knowledge, this is the first study where HbA1c has been evaluated in POTS. We have previously shown that the median time from symptom onset to POTS diagnosis was 3 (IQR 1–10) years, and the median time from diagnosis to study inclusion, when HbA1c was measured, was 3 (IQR 1–7) years^56^. Thus, despite several years with a clinically confirmed POTS diagnosis, which may be associated with altered insulin dynamics, HbA1c was normal.

There were no differences in fasting and non-fasting levels of leptin, glucagon, GIP, GLP-1, and PYY between POTS and controls. Metabolic gut hormones are secreted by entero-endocrine cells throughout the GI tract as a response to ingested food, and the levels and timing of the hormonal release depend on luminal food composition, including amounts of ingested calories^17,57^. When secreted, these hormones affect motility, secretion, permeability, and visceral sensibility of the gut locally and by interaction with afferent and efferent fibers of the enteric and autonomic nervous system, and finally the central nervous system^17^. Once secreted, many of the metabolic hormones are rapidly degraded and levels in serum or plasma are low between meals^11,58^. In patients with IBS, studies have shown altered expression of entero-endocrine cells throughout the gut^17,59^. However, dietary treatment or fecal microbiota transplant in IBS normalize this expression, suggesting an intricate interplay between diet, gut microbiota, entero-endocrine cells, and gut hormones^60^. A recent study showed reduced GI symptoms and increased fasting levels of GLP-1 in IBS patients following a 12-week low FODMAP diet^61^, and a negative correlation has been found between circulating levels of GLP-1 and abdominal pain in constipation-predominant IBS^13^. On the contrary, non-fasting levels of GIP, GLP-1, and PYY were unaltered after a 4-week treatment with a starch and sucrose reduced diet in IBS patients, and without correlations to GI symptoms^18^. In the present study, there were no significant correlations between levels of non-fasting hormones and GI symptoms in POTS.

The strength of the present study is that we have used two large, well-defined cohorts of POTS patients from a tertiary center, which were compared with matched healthy controls. All patients were properly diagnosed by tilt test before inclusion. We have also obtained data on GI symptoms with validated symptom scores and patients and control participants were clinically examined. We have used clinically validated methods for analysis of routine blood samples, and Hb and erc-MCV were measured to exclude common pitfalls in the interpretation of HbA1c.

There are several limitations of the study. First, there was a substantial lack of data regarding BMI levels in the fasting cohort but also lack of data regarding waist circumference. Even though leptin levels were comparable between groups, the distribution of adipose tissue was unknown. Waist circumference would have been more informative regarding potential explanations to the findings regarding C-peptide/insulin and BP measures^62^. Another important limitation was that we did not measure fasting plasma glucose at the time of inclusion when the fasting blood samples were obtained during 2017–2019. Therefore, we cannot draw any conclusions regarding insulin resistance in POTS, despite findings of normal HbA1c. Some patients were unable to abstain from cardiovascular medicines 48 h before the active standing test, and fasting blood sampling. Medications could possibly affect the hormonal levels, as mentioned above in the discussion section. In the sensitivity analysis, we decided to keep one patient who reported use of Venlafaxine and two patients who reported use of Progestin-only contraceptives to avoid losing power, although these medicines may have a minor effect on glucose metabolism^32,33^. Another limitation is that current comorbidities were not reported in the fasting cohort. In the non-fasting cohort, patients were allowed to continue with their medicines, where several medicines may have potential effects on hormonal levels. Moreover, non-fasting measurement of metabolic gut hormonal levels is tricky to interpret since these hormones are released as response to ingested nutrients and we did not obtain food diary immediately before blood sampling. However, blood samples were taken approximately during the same time span of the day in both POTS and controls. We did not measure glomerular filtration rate or hepatic function, which are important in degradation and elimination of gut hormones^63,64^. The analysis of cortisol was added after the results regarding insulin and its association with BP in POTS were revealed, to find potential explanations to this finding. Therefore, all results regarding cortisol levels in POTS must be interpreted cautiously. Finally, some of the metabolic gut hormonal levels were low in the present study, thus, the precision of the Mesoscale Discovery assay may be less accurate.

## Conclusion

According to the present study, there is no evidence of long-term disturbances in glucose metabolism in POTS. However, fasting C-peptide/insulin levels are positively correlated with blood pressure in POTS – an association that is not observed in healthy controls – but the underlying mechanisms remain unclear. Together, these findings suggest a potential link between insulin-related pathways and blood pressure regulation in POTS. Future studies are warranted to further investigate insulin dynamics in POTS, including longitudinal studies, to evaluate whether incidence of hypertension and cardiovascular disease is impacted by the presence of POTS.

## Supplementary Information

Below is the link to the electronic supplementary material.

Supplementary Material 1

## Data Availability

All the relevant data that cannot compromise patient confidentiality, and that support the findings of this study, are within the manuscript and its supporting information files. Further data from this study are available from the corresponding author, (HT), upon reasonable request.
