# Supplementary material for: Gut hormones in POTS and their relation to hemodynamic parameters and gastrointestinal symptoms
Source: Sci Rep. 2026 May 19;16:15514. doi: 10.1038/s41598-026-52963-0 (PMC13187148; doi:10.1038/s41598-026-52963-0)
Supplement: Supplementary file 1 — Supplementary Material 1 [file 41598_2026_52963_MOESM1_ESM.docx]

**Supplementary Table S1.** Comparisons of metabolic gut hormonal levels in EDTA plasma and serum

| Sample No | C-peptid  (pg/mL) | GIP total  (pg/mL) | GLP-1  (pM) | Glucagon  (pM) | Insulin  (µU/mL) | Leptin  (pg/mL) | PYY  (pg/mL) |
| --- | --- | --- | --- | --- | --- | --- | --- |
| 1 Serum | 844 | 91.2 | 15.8 | 13.4 | 5.6 | 9,146 | 78.5 |
| 1 EDTA | 752 | 217.7 | 16.6 | 15.0 | 6.0 | 8,588 | 78.2 |
| 2 Serum | 3,074 | 643.7 | 21.3 | 20.5 | 44.4 | 23,526 | 102 |
| 2 EDTA | 3,391 | 1,387 | 27.7 | 20.2 | 49.4 | 23,279 | 108 |
| 3 Serum | 2,064 | 84.7 | 15.8 | 5.2 | 23.5 | 30,735 | 65.8 |
| 3 EDTA | 2,078 | 99.0 | 16.8 | 18.2 | 23.7 | 28,401 | 71.5 |
| 4 Serum | 823 | 36.4 | 8.1 | 10.0 | 7.3 | 3,082 | 30.6 |
| 4 EDTA | 829 | 72.4 | 8.8 | 10.9 | 8.2 | 3,135 | 31.4 |

Four of the samples were analyzed in both EDTA plasma and serum with good agreement on all hormones except GIP where serum levels were significantly lower in serum compared to EDTA plasma. GIP: glucose-dependent insulinotropic peptide; GLP-1: glucagon-like peptide-1; PYY: peptide YY.

**Supplementary Table S2.** Average intra-assay and inter-assay coefficients of variance (CV) from hormonal analyses.

|  | **Concentration** | **CV intra-assay (%)** | **CV inter-assay (%)** |
| --- | --- | --- | --- |
| **C-peptide** (n=6) | High (4,610 pg/mL) | 4.5 | 10.4 |
|  | Medium (1,890 pg/mL) | 2.9 | 12.9 |
|  | Low (871 pg/mL) | 4.1 | 14.8 |
| **GIP, total** (n=6) | High (3,370 pg/mL) | 8.3 | 10.7 |
|  | Medium (622 pg/mL) | 4.0 | 13.7 |
|  | Low (158 pg/mL) | 11.1 | 16.9 |
| **GLP-1, total** (n=6) | High (417 pM) | 2.7 | 7.5 |
|  | Medium (93 pM) | 2.5 | 9.5 |
|  | Low (25 pM) | 3.6 | 12.8 |
| **Glucagon** (n=6) | High (56 pM) | 4.5 | 11.9 |
|  | Medium (12 pM) | 4.0 | 13.4 |
|  | Low (2.5 pM) | 11.5 | 17.1 |
| **Insulin**  (n=6) | High (347 uU/mL) | 3.4 | 16.8 |
|  | Medium (89 uU/mL) | 2.6 | 15.3 |
|  | Low (15 uU/mL) | 2.7 | 20.8 |
| **Leptin**  (n=6) | High (24300 pg/mL) | 5.2 | 10.7 |
|  | Medium (4210 pg/mL) | 2.9 | 10.7 |
|  | Low (841 pg/mL) | 5.3 | 16.1 |
| **PYY**  (n=6) | High (1020 pg/mL) | 3.9 | 8.9 |
|  | Medium (274 pg/mL) | 3.2 | 12.1 |
|  | Low (82 pg/mL) | 4.7 | 16.6 |

Data obtained from six different samples, and at three different concentrations for each hormone, obtained by The Mesoscale Discovery. Abbreviations: GIP=gastrointestinal peptide; GLP-1=glucagon-like peptide-1; PYY=peptide YY.

**Supplementary Table S3.** Drugs regularly taken in the fasting POTS cohort

| **Cardiovascular drugs** | **POTS**  **N=42** |
| --- | --- |
| I(f) receptor antagonists | 7 |
| Selective Beta-1 blockers | 11 |
| Non-selective Betablockers | 7 |
| Norepinehrine prodrugs | 10 |
| Cholinesterase inhibitors | 2 |
| Mineral corticoids | 0 |
| Short acting nitroglycerine | 3 |
| Calcium channel antagonists | 2 |
| Angiotensin receptor antagonists | 1 |
| Aldosterone antagonists | 1 |
| Alpha-1 adrenergic agonists | 6 |
| Central nervous system stimulants | 6 |
| **Asthma and allergy** |  |
| Histamine H1 antagonists | 8 |
| Inhaled β2-agonists | 5 |
| Leukotriene receptor agonists | 1 |
| Inhaled steroids | 3 |
| Sodium Cromoglicate | 2 |
| Nasal steroids | 1 |
| Inhaled ipratropiumbromide | 1 |
| Oral budesonide | 1 |
| Eye drops | 1 |
| Oral systemic steroids, taken on demand | 2 |
| **Hormonal drugs** |  |
| Combined oral hormonal contraceptives | 4 |
| Progestin-only contraceptives | 3 |
| Thyroid hormones | 2 |
| **Pain killers** |  |
| Acetaminophen | 5 |
| Opioids and opioid-like drugs, taken on demand | 2 |
| **Non-steroidal Anti-Inflammatory Drugs** | 3 |
| Muscle relaxing agents | 2 |
| Gabapentinoids | 3 |
| Spasmolytics Papaverin on demand | 1 |
| Baclofen | 1 |
| **Vitamin and mineral supplementation** |  |
| B12 (oral or injected) | 2 |
| D-vitamins | 1 |
| Folic acid | 3 |
| Potassium chloride | 1 |
| **Antidepressants and mood stabilizers** |  |
| Serotonin and noradrenaline reuptake inhibitors | 4 |
| Noradrenaline reuptake inhibitors | 1 |
| Antiepileptic drugs | 1 |
| Tricyclic antidepressants | 3 |
| Other antidepressants | 1 |
| Mirtazapin, taken on demand | 1 |
| **Gastrointestinal drugs** |  |
| Histamine H2-antagnoist | 1 |
| Proton pump inhibitors | 3 |
| 5HT3-antagonists | 3 |
| 5HT4-agonist | 1 |
| 5-ASA | 1 |
| Pancreatic enzymes | 1 |
| Bile acid sequestrants | 1 |
| Anti-flatular agensts | 2 |
| **Sleeping pills** |  |
| Melatonin | 4 |
| Benzodiazepine-like | 1 |
| Others | 3 |
| **Tranquilizers** |  |
| Non-benzodiazepines | 1 |
| Benzodiazepines | 1 |
| **Miscellaneous** |  |
| Triptans | 1 |
| Statins | 1 |
| Metformin | 1 |
| Metotrexat | 1 |
| Pyridoxin | 1 |
| Adrenaline, taken on demand | 1 |

**Supplementary Table S4**. Fasting levels of serum metabolic gut hormones and morning plasma cortisol in POTS and healthy controls after sensitivity analysis

|  | **POTS (n=27)** | **Controls (n=41)** | **p-value** |
| --- | --- | --- | --- |
| **Insulin (µU/mL)** | 15.3 (10.9–18.4) | 11.9 (9.1–15.7)^a^ | 0.061 |
| **C-peptide (pg/mL)** | 1638 (1145–1982) | 1315 (1084–1677)^a^ | 0.197 |
| **Glucagon (pM)** | 15.0 (10.4–20.8) | 12.7 (10.0–16.5)^a^ | 0.237 |
| **GLP-1 (pM)** | 8.8 (7.6–10.9) | 8.6 (7.1–10.3)^a^ | 0.347 |
| **GIP total (pg/mL)** | 36.9 (28.4–57.3) | 32.3 (23.8–62.2)^b^ | 0.334 |
| **PYY (pg/mL)** | 47.7 (35.1–71.9) | 47.5 (33.3–57.3)^a^ | 0.574 |
| **Leptin (pg/mL)** | 27488 (10741–44670) | 19922 (10798–31825)^a^ | 0.266 |
| **Cortisol (nL)** | 404 (273–507) | 404 (297–570) | 0.334 |

Values presented as median (interquartile range). Comparative analyses were performed with Mann Whitney-U test. A p-value <0.05 was considered statistically significant. POTS: postural orthostatic tachycardia syndrome; GIP: glucose-dependent insulinotropic polypeptide; GLP-1: glucagon-like peptide-1; PYY: peptide YY.

^a^1 missing value

^b^2 missing values

**Supplementary Table S5.** Correlations between fasting metabolic gut hormonal levels and hemodynamic parameters after sensitivity analysis

|  | **C-peptide (pg/mL)** | | | **Insulin (µU/mL)** | | | **Leptin (pg/mL)** | | |
| --- | --- | --- | --- | --- | --- | --- | --- | --- | --- |
|  | POTS (n=27) | Control (n=40) | Fisher r to z | POTS (n=27) | Control (n=40) | Fisher r to z | POTS (n=27) | Control (n=40) | Fisher r to z |
| **SBP_sup_** | r=0.538  p=0.004  q=0.005 | r=0.192  p=0.235  q=0.554 | z=1.873  p=0.061  q=0.070 | r=0.583  p=0.001  q=0.002 | r=0.110  p=0.500  q=0.863 | z=2.123  p=0.034  q=0.039 | r=0.482  p=0.011  q=0.132 | r=0.164  p=0.313  q=0.360 | z=1.374  p=0.169 |
| **DBP_sup_** | r=0.488  p=0.010  q=0.010 | r=0.256  p=0.110  q=0.554 | z=1.036  p=0.300  q=0.300 | r=0.472  p=0.013  q=0.020 | r=0.261  p=0.104  q=0.572 | z=0.937  p=0.349  q=0.349 | r=0.312  p=0.113  q=0.206 | r=0.334  p=0.035  q=0.086 | z=-0.094  p=0.925 |
| **HR_sup_** | r=0.226  p=0.256  q=0.303 | r=0.178  p=0.272  q=0.554 |  | r=0.307  p=0.120  q=0.160 | r=0.186  p=0.250  q=0.706 |  | r=0.099  p=0.624  q=0.624 | r=0.333  p=0.036  q=0.086 |  |
| **SBP_1_** | r=0.692^a^  p<0.001  q=0.008 | r=0.086  p=0.599  q=0.719 | z=2.802  p=0.005  q=0.024 | r=0.821^a^  p<0.001  q=0.002 | r=-0.006  p=0.972  q=0.972 | z=4.267  p<0.001  q=0.002 | r=0.297^a^  p=0.159  q=0.239 | r=0.192  p=0.236  q=0.360 | z=0.409  p=0.682 |
| **DBP_1_** | r=0.618^a^  p=0.001  q=0.015 | r=0.160  p=0.323  q=0.554 | z=2.051  p=0.040  q=0.064 | r=0.668^a^  p=0.003  q=0.002 | r=0.035  p=0.828  q=0.903 | z=2.826  p=0.004  q=0.005 | r=0.398^a^  p=0.054  q=0.206 | r=0.404  p=0.010  q=0.040 | z=-0.026  p=0.979 |
| **ΔHR_1_** | r=-0.088^a^  p=0.684  q=0.341 | r=0.169  p=0.298  q=0.554 |  | r=-0.056^a^  p=0.794  q=0.866 | r=0.170  p=0.294  q=0.706 |  | r=-0.115^a^  p=0.593  q=0.624 | r=-0.163  p=0.314  q=0.360 |  |
| **SBP_3_** | r=0.633^b^  p<0.001  q=0.003 | r=0.123  p=0.451  q=0.651 | z=2.313  p=0.021  q=0.056 | r=0.763^b^  p<0.001  q=0.002 | r=-0.075  p=0.647  q=0.863 | z=4.006  p<0.001  q=0.002 | r=0.339^b^  p=0.097  q=0.206 | r=0.181  p=0.263  q=0.360 | z=0.631  p=0.528 |
| **DBP_3_** | r=0.554^b^  p=0.004  q=0.003 | r=0.113  p=0.488  q=0.651 | z=1.897  p=0.058  q=0.070 | r=0.663^b^  p<0.001  q=0.002 | r=-0.057  p=0.725  q=0.870 | z=3.177  p=0.001  q=0.002 | r=0.319^b^  p=0.120  q=0.206 | r=0.440  p=0.005  q=0.040 | z=-0.536  p=0.599 |
| **ΔHR_3_** | r=0.032^b^  p=0.878  q=0.759 | r=0.238  p=0.139  q=0.554 |  | r=-0.003^b^  p=0.987  q=0.987 | r=-0.236  p=0.143  q=0.572 |  | r=0.205^b^  p=0.325  q=0.433 | r=-0.158  p=0.330  q=0.360 |  |
| **SBP_5_** | r=0.646^b^  p<0.001  q=0.003 | r=0.023^c^  p=0.888  q=0.969 | z=2.754  p=0.006  q=0.024 | r=0.712^b^  p<0.001  q=0.002 | r=-0.092^c^  p=0.577  q=0.863 | z=3.634  p<0.001  q=0.002 | r=0.389^b^  p=0.055  q=0.206 | r=0.228^c^  p=0.164  q=0.328 | z=0.650  p=0.509 |
| **DBP_5_** | r=0.512^b^  p=0.009  q=0.015 | r=-0.001^c^  p=0.995  q=0.995 | z=2.093  p=0.036  q=0.064 | r=0.651^b^  p<0.001  q=0.002 | r=-0.107^c^  p=0.517  q=0.863 | z=3.268  p=0.001  q=0.002 | r=0.358^b^  p=0.079  q=0.206 | r=0.408^c^  p=0.010  q=0.040 | z=-1.185  p=0.236 |
| **ΔHR_5_** | r=0.082^b^  p=0.696  q=0.759 | r=0.365^c^  p=0.022  q=0.264 |  | r=-0.138^b^  p=0.511  q=0.613 | r=0.414^c^  p=0.009  q=0.108 |  | r=0.112^b^  p=0.594  q=0.624 | r=0.018^c^  p=0.915  q=0.915 |  |

Correlations performed with Spearman’s test in 27 patients with POTS and 40 controls. Differences in correlations between groups were calculated with Fisher’s r-to-z test. Q = P-values adjusted for false discovery rate according to the Benjamini-Hochberg procedure. A q-value <0.05 was considered significant. POTS: postural orthostatic tachycardia syndrome; SBP: systolic blood pressure; DBP: diastolic blood pressure; HR: heart rate.

^a^3 missing

^b^2 missing

^c^1 missing

**Supplementary Table S6.** Correlations between fasting levels of metabolic gut hormones and hemodynamic parameters during active standing tests in POTS and controls

|  | **GIP (pg/mL)** | | **GLP-1 (pM)** | | **Glucagon (pM)** | | **PYY (pg/mL)** | |
| --- | --- | --- | --- | --- | --- | --- | --- | --- |
|  | POTS | Control | POTS | Control | POTS | Control | POTS | Control |
| **SBP_sup_** | r=0.001^a^  p=0.995 | r=-0.077^a^  p=0.642  q=0.808 | r=0.028^a^  p=0.862 | r=0.078  p=0.633  q=0.839 | r=-0.002^a^  p=0.991 | r=-0.078  p=0.632  q=0.632 | r=-0.074^a^  p=0.644  q=0.859 | r=-0.192  p=0.236  q=0.566 |
| **DBP_sup_** | r=-0.062^a^ p=0.700 | r=0.056^a^  p=0.734  q=0.808 | r=-0.031^a^  p=0.845 | r=-0.063  p=0.699  q=0.839 | r=0.082^a^  p=0.608 | r=-0.228  p=0.157  q=0.236 | r=-0.180^a^  p=0.259  q=0.518 | r=-0.107  p=0.518  q=0.622 |
| **HR_sup_** | r=-0.252^a^  p=0.112 | r=-0.157^a^  p=0.339  q=0.808 | r=-0.246^a^  p=0.121 | r=-0.223  p=0.166  q=0.398 | r=-0.062^a^  p=0.712 | r=-0.244  p=0.130  q=0.236 | r=-0.325^a^  p=0.038  q=0.384 | r=-0.235  p=0.144  q=0.431 |
| **SBP_1_** | r=0.053^b^  p=0.751 | r=0.115^a^  p=0.486  q=0.808 | r=0.172^b^  p=0.303 | r=-0.108  p=0.506  q=0.839 | r=0.291^b^  p=0.077 | r=-0.235  p=0.145  q=0.236 | r=0.033^b^  p=0.842  q=0.919 | r=-0.142  p=0.381  q=0.572 |
| **DBP_1_** | r=-0.003^b^  p=0.988 | r=-0.020^a^  p=0.902  q=0.902 | r=0.043^b^  p=0.798 | r=-0.302  p=0.059  q=0.236 | r=0.103^b^  p=0.538 | r=-0.269  p=0.093  q=0.236 | r=-0.017^b^  p=0.922  q=0.922 | r=-0.165  p=0.309  q=0.572 |
| **ΔHR_1_** | r=-0.160^b^  p=0.338 | r=-0.422^a^  p=0.007  q=0.084 | r=-0.291^b^  p=0.076 | r=0.007  p=0.965  q=0.965 | r=-0.048^b^  p=0.774 | r=0.251  p=0.119  q=0.236 | r=-0.195^b^  p=0.242  q=0.518 | r=0.000  p=0.998  q=0.998 |
| **SBP_3_** | r=0.057^b^  p=0.735 | r=0.057^a^  p=0.728  q=0.808 | r=0.109^b^  p=0.513 | r=-0.018  p=0.910  q=0.965 | r=0.159^b^  p=0.341 | r=-0.081  p=0.619  q=0.632 | r=0.045^b^  p=0.789  q=0.919 | r=-0.145  p=0.373  q=0.572 |
| **DBP_3_** | r=-0.035^b^  p=0.833 | r=-0.055^a^  p=0.741  q=0.808 | r=-0.065^b^  p=0.700 | r=-0.302  p=0.058  q=0.236 | r=0.084^b^  p=0.616 | r=-0.119  p=0.466  q=0.560 | r=-0.096^b^  p=0.567  q=0.859 | r=-0.107  p=0.509  q=0.622 |
| **ΔHR_3_** | r=-0.160^b^  p=0.337 | r=-0.350^a^  p=0.029  q=0.174 | r=-0.296^b^  p=0.072 | r=0.094  p=0.564  q=0.839 | r=-0.116^b^  p=0.488 | r=0.335  p=0.034  q=0.204 | r=-0.303^b^  p=0.064  q=0.384 | r=0.068  p=0.677  q=0.739 |
| **SBP_5_** | r=-0.078^c^  p=0.647 | r=-0.109^d^  p=0.517  q= | r=0.215^c^  p=0.200 | r=-0.155^a^  p=0.345  q=0.690 | r=0.094^c^  p=0.579 | r=-0.219^a^  p=0.185  q=0.247 | r=-0.080^c^  p=0.639  q=0.859 | r=-0.320^a^  p=0.047  q=0.264 |
| **DBP_5_** | r=-0.034^c^  p=0.840 | r=-0.104^d^  p=0.533  q=0.808 | r=-0.045^c^  p=0.790 | r=-0.350^a^  p=0.029  q=0.236 | r=0.008^c^  p=0.961 | r=-0.307^a^  p=0.058  q=0.232 | r=-0.211^c^  p=0.211  q=00.518 | r=-0.331^a^  p=0.039  q=0.264 |
| **ΔHR_5_** | r=-0.125^c^  p=0.459 | r=-0.263^d^  p=0.110  q=0.440 | r=-0.157^c^  p=0.353 | r=0.266^a^  p=0.101  q=0.303 | r=0.024^c^  p=0.887 | r=0.355^a^  p=0.027  q=0.204 | r=-0.240^c^  p=0.153  q=0.518 | r=0.298^a^  p=0.066  q=0.264 |

Correlations performed with Spearman’s test in 42 patients with POTS and 40 controls. Q = P-values adjusted for false discovery rate according to the Benjamini-Hochberg procedure. A q-value < 0.05 was considered significant. SBP: systolic blood pressure; DBP: diastolic blood pressure; HR: heart rate; GIP: glucose-dependent insulinotropic peptide; GLP-1: glucagon-like peptide-1; PYY: peptide YY.

^a^1 missing value

^b^4 missing values
^c^5 missing values

^d^2 missing values

**Supplementary Table S7.** Correlations between fasting plasma cortisol levels and C-peptide, insulin and hemodynamic parameters

|  | **Morning plasma cortisol (nmol/L)** | |
| --- | --- | --- |
|  | POTS (n=42) | Controls (n=41) |
| C-peptide (pg/mL) | r=-0.282  p=0.071  q=0.355 | r=0.397^a^  p=0.011  q=0.090 |
| Insulin (µU/mL) | r=-0.170  p=0.281  q=0.430 | r=0.373^a^  p=0.018  q=0.090 |
| SBP_supine_ | r=-0.213^a^  p=0.182  q=0.430 | r=0.283  p=0.073  q=0.215 |
| DBP_supine_ | r=-0.362^a^  p=0.020  q=0.200 | r=0.207  p=0.194  q=0.323 |
| SBP_1min_ | r=-0.112^b^  p=0.502  q=0.628 | r=0.271  p=0.086  q=0.215 |
| DBP_1min_ | r=-0.172^b^  p=0.301  q=0.430 | r=0.164  p=0.305  q=0.436 |
| SBP_3min_ | r=-0.088^b^  p=0.598  q=0.647 | r=0.232  p=0.145  q=0.290 |
| DBP_3min_ | r=-0.077^b^  p=0.647  q=0.647 | r=0.128  p=0.427  q=0.534 |
| SBP_5min_ | r=-0.178^c^  p=0.293  q=0.430 | r=0.068^a^  p=0.675  q=0.750 |
| DBP_5min_ | r=-0.175^c^  p=0.301  q=0.430 | r=0.047^a^  p=0.775  q=0.775 |

Correlation analyses were performed using Spearman’s correlation analysis. Q = P-values adjusted for false discovery rate according to the Benjamini-Hochberg procedure. A q-value <0.05 was considered statistically significant. SBP: systolic blood pressure; DBP: diastolic blood pressure.

^a^1 missing

^b^3 missing

^c^5 missing

**Supplementary Table S8.** Self-reported comorbidities in the non-fasting cohort

| **Comorbidities** |  | **POTS (N=43)** | **Healthy controls (N=52)** |
| --- | --- | --- | --- |
| **Gastrointestinal disorders** |  |  |  |
|  | Irritable bowel syndrome | 12 |  |
|  | Obstipation | 3 |  |
|  | Gastroparesis | 3 |  |
|  | Celiac disease | 2 |  |
|  | Inflammatory bowel disease | 2 |  |
|  | Microscopic colitis | 2 |  |
|  | Dyspepsia | 2 | 1 |
|  | Polyposis syndrome | 1 |  |
|  | Lactose intolerance | 1 |  |
|  | Gastroesofageal reflux disorder/Peptic ulcer disease | 1 |  |
|  | Diverticulitis | 1 |  |
|  | Cholelithiasis | 1 |  |
| **Rheumathological and musculosceletal disorders** |  |  |  |
|  | HSD/EDS | 12 |  |
|  | Fibromyalgia | 2 |  |
|  | Lumbago/herniated disc/scoliosis | 2 |  |
|  | Psoriasis arthritis | 1 |  |
|  | Raynaud syndrom | 1 |  |
|  | Whiplash injury | 1 |  |
|  | Back injury | 1 |  |
|  | Meniscus injury | 1 |  |
| **Psychiatric diseases** |  |  |  |
|  | Neuropsychiatric disorders (ADHD/ADD/Autism spectrum disorders) | 6 |  |
|  | Depression and anxiety disorders | 4 |  |
|  | Eating disorders | 1 |  |
|  | Bipolar disorder | 1 |  |
| **Asthma and allergy** |  |  |  |
|  | Asthma | 9 | 1 |
|  | Allergy | 2 | 3 |
| **Gynaecological disorders** |  |  |  |
|  | Endometriosis | 5 |  |
|  | Myoma | 1 |  |
|  | Polycysic ovarian syndrome | 1 |  |
|  | Premenstrual dysphoric disorder |  |  |
| **Neurological disorders** |  |  |  |
|  | Migraine | 7 | 1 |
|  | Tinnitus | 1 | 1 |
| **Endocrine disorders** |  |  |  |
|  | Thyroid disease (hypothyroidism, goiter, graves thyrotoxicosis) | 6 |  |
|  | Diabetes Mellitus II | 1 |  |
| **Cardiovascular diseases** |  |  |  |
|  | Sick sinus syndrome | 2 |  |
|  | Inappropriate sinus tachycardia | 2 |  |
|  | Pulmonary embolism | 1 |  |
| **Skin diseases** |  |  |  |
|  | Rosacea | 1 | 1 |
|  | Lichen sclerosus | 1 |  |
|  | Eczema |  | 2 |
|  | Psoriasis |  | 1 |
| **Disorders of the urinary tract** |  |  |  |
|  | Renal disease | 1 |  |
|  | Urinary bladder dysfunction | 1 |  |
| **Miscellaneous** |  |  |  |
|  | Myalgic encephalomyelits | 5 |  |
|  | Mast cell activation | 4 |  |
|  | Post-Covid | 3 |  |
|  | Exhaustion disorder | 1 |  |

ADHD=Attention-Deficit/Hyperactivity Disorder, ADD=Attention-Deficit Disorder, HSD/EDS=hypermobile spectrum disorder/Ehlers-Danlos syndrome

**Supplementary Table S9.** Pharmacological medications in the non-fasting POTS cohort

|  |  | **POTS**  **N=43** | **Healthy controls N=52** |
| --- | --- | --- | --- |
| **Cardiovascular and POTS-related drugs** |  |  |  |
|  | Antihypotensive agents | 17 | 0 |
|  | Ivabradine I(f) receptor inhibitors | 16 | 0 |
|  | Beta blockers | 13 | 0 |
|  | Central stimulating agents | 8 | 0 |
|  | Cholinesterase inhibitors | 6 | 0 |
|  | Mineral corticoids | 3 | 0 |
|  | Short acting nitroglycerine | 3 | 0 |
|  | Calcium channel blockers | 2 | 0 |
|  | Angiotensin receptor blockers | 1 | 0 |
| **Asthma and allergy** |  |  |  |
|  | Histamine H1-blockers | 13 | 1 |
|  | Inhaled β2-agonists | 7 | 0 |
|  | Leukotriene receptor agonists | 5 | 0 |
|  | Inhaled steroids | 4 | 0 |
|  | Sodium Cromoglicate | 3 | 0 |
|  | Nasal steroids | 2 | 1 |
|  | Inhaled ipratropiumbromide | 1 | 0 |
|  | Oral budesonide | 1 | 0 |
|  | Eye drops | 0 | 1 |
|  | Topical steroids | 0 | 1 |
| **Hormonal drugs** |  |  |  |
|  | Combined hormonal contraceptives | 8 | 2 |
|  | Progesterone | 6 | 1 |
|  | Thyroid hormones | 5 | 0 |
|  | GLP-1-analogs | 1 | 0 |
| **Pain killers** |  |  |  |
|  | Acetaminophen | 6 | 1 |
|  | Opioids and opioid-like drugs | 5 | 0 |
|  | **Non-steroidal Anti-Inflammatory Drugs** | 3 | 1 |
|  | Muscle relaxing agents | 2 | 0 |
|  | Gabapentinoids | 2 | 0 |
|  | Spasmolytics | 1 | 0 |
| **Vitamin and mineral supplementation** |  |  |  |
|  | B12 (oral or injected) | 6 | 0 |
|  | D-vitamins | 4 | 0 |
|  | Multivitamins | 3 | 1 |
|  | Folic acid | 2 | 0 |
|  | Potassium chloride | 1 | 0 |
|  | Magnesium | 1 | 0 |
|  | Sodium chloride tablets | 1 | 0 |
| **Antidepressants and mood stabilizers** |  |  |  |
|  | Selective serotonin reuptake inhibitor | 7 | 1 |
|  | Serotonin and noradrenaline reuptake inhibitors | 3 | 0 |
|  | Noradrenaline reuptake inhibitors | 3 | 0 |
|  | Antiepileptic drugs | 2 | 0 |
|  | Tricyclic antidepressants | 2 | 0 |
|  | Other antidepressants | 1 | 0 |
| **Gastrointestinal drugs** |  |  |  |
|  | Histamine H2-blockers | 7 | 0 |
|  | Proton pump inhibitors | 2 | 1 |
|  | 5HT3-antagonists | 2 | 0 |
|  | 5HT4-antagonists | 2 | 0 |
|  | Laxatives | 1 | 0 |
|  | Bulking agents | 0 | 0 |
| **Sleeping pills** |  |  |  |
|  | Melatonin | 4 | 1 |
|  | Benzodiazepine-like | 2 | 1 |
|  | Others | 3 | 0 |
| **Tranquilizers** |  |  |  |
|  | Non-benzodiazepines | 3 | 0 |
|  | Benzodiazepines | 1 | 0 |
| **Miscellaneous** |  |  |  |
|  | Triptans | 2 | 0 |
|  | Statins | 2 | 0 |
|  | TNF-α antagonist | 1 | 0 |
|  | Direct oral anticoagulants | 1 | 0 |

GLP=glucagon like peptide. PMDD=premenstrual dysphoric disorder. TNF=tumor necrosis factor.

**Supplementary Table S10.** Correlations between HbA1c levels and non-fasting metabolic gut hormones in POTS and controls

|  | **HbA1c (mmol/mol)** | |
| --- | --- | --- |
|  | POTS (N=43) | Controls (N=52) |
| C-peptide (pg/mL) | r=0.118^a^  p=0.480 | r=0.243^b^  p=0.141 |
| GIP (pg/mL) | r=0.040^a^  p=0.814 | r=0.296^b^  p=0.071 |
| GLP-1 (pM) | r=0.132^a^  p=0.428 | r=-0.055^b^  p=0.742 |
| Glucagon (pM) | r=0.267^a^  p=0.105 | r=0.148^b^  p=0.376 |
| Insulin (µU/mL) | r=0.131^a^  p=0.432 | r=0.189^b^  p=0.255 |
| Leptin (pg/mL) | r=0.062^a^  p=0.711 | r=0.130^b^  p=0.437 |
| PYY (pg/mL) | r=0.102^a^  p=0.541 | r=0.072^b^  p=0.668 |

Correlation analyses were performed using Spearman’s correlation analysis. A p-value <0.05 was considered statistically significant. GIP: glucose-dependent insulinotropic peptide; GLP-1: glucagon-like peptide-1; PYY: peptide YY.

^a^5 missing

^b^14 missing

**Supplementary Table S11.** Correlations between BMI and abdominal symptoms in the non-fasting POTS cohort

|  | **BMI (kg/m^2^)** |
| --- | --- |
| Abdominal pain | r=0.054  p=0.732 |
| Diarrhea | r=0.089  p=0.573 |
| Constipation | r=0.209  p=0.183 |
| Bloating and flatulence | r=0.102  p=0.521 |
| Vomiting and nausea | r=-0.133  p=0.402 |
| Psychological well-being | r=0.067  p=0.674 |
| Symptoms’ influence on daily life | r=0.052  p=0.742 |
| Total IBS-SSS | r=0.109  p=0.511 |

Correlation analyses were performed using Spearman’s correlation analysis on 42 of 43 patients with POTS. A p-value <0.05 was considered statistically significant.

**Supplementary Table S12.** Correlations between non-fasting metabolic gut hormonal levels and gastrointestinal symptoms in POTS and controls

|  | **C-peptide (pg/mL)** | | **GIP (pg/mL)** | | **GLP-1 (pM)** | | **Glucagon (pM)** | | **Insulin (µU/mL)** | | **Leptin (pg/mL)** | | **PYY (pg/mL)** | |
| --- | --- | --- | --- | --- | --- | --- | --- | --- | --- | --- | --- | --- | --- | --- |
|  | POTS | Control | POTS | Control | POTS | Control | POTS | Control | POTS | Control | POTS | Control | POTS | Control |
| **Abdominal pain** | r=-0.156^a^  p=0.331 | r=0.025  p=0.859 | r=-0.141^a^  p=0.379  q=0.656 | r=0.106  p=0.455 | r=-0.052^a^  p=0.748 | r=-0.026  p=0.857 | r=-0.094^a^  p=0.559 | r=0.036  p=0.800 | r=-0.126^a^  p=0.432 | r=-0.083  p=0.557 | r=0.131^a^  p=0.414 | r=-0.052  p=0.714  q=0.714 | r=-0.262^a^  p=0.097  q=0.267 | r=-0.053  p=0.710 |
| **Diarrhea** | r=0.036^a^  p=0.825 | r=0.025  p=0.860 | r=0.081^a^  p=0.615  q=0.765 | r=0.063  p=0.656 | r=-0.055^a^  p=0.735 | r=-0.106  p=0.454 | r=-0.208^a^  p=0.193 | r=-0.089  p=0.528 | r=0.050^a^  p=0.758 | r=-0.069  p=0.626 | r=0.015^a^  p=0.924 | r=0.152  p=0.284  q=0.325 | r=-0.082^a^  p=0.610  q=0.610 | r=-0.049  p=0.728 |
| **Constipation** | r=0.126^a^  p=0.434 | r=-0.047  p=0.738 | r=-0.028^a^  p=0.860  q=0.860 | r=-0.036  p=0.802 | r=0.187^a^  p=0.243 | r=-0.083  p=0.560 | r=0.294^a^  p=0.062 | r=-0.004  p=0.979 | r=0.088^a^  p=0.585 | r=-0.113  p=0.424 | r=0.291^a^  p=0.065 | r=0.181  p=0.199  q=0.325 | r=-0.128^a^  p=0.425  q=0.572 | r=-0.036  p=0.800 |
| **Bloating and flatulence** | r=0.118^a^  p=0.462 | r=0.061^b^  p=0.669 | r=-0.180^a^  p=0.260  q=0.656 | r=-0.038^b^  p=0.792 | r=-0.136^a^  p=0.398 | r=-0.060^b^  p=0.674 | r=-0.260^a^  p=0.100 | r=-0.028^b^  p=0.845 | r=0.136^a^  p=0.397 | r=0.031^b^  p=0.831 | r=0.124^a^  p=0.438 | r=0.241^b^  p=0.088  q=0.235 | r=-0.186^a^  p=0.245  q=0.490 | r=0.050^b^  p=0.726 |
| **Vomiting and nausea** | r=-0.086^a^  p=0.594 | r=0.076  p=0.595 | r=-0.132^a^  p=0.410  q=0.656 | r=0.175  p=0.216 | r=-0.052^a^  p=0.749 | r=0.061  p=0.665 | r=-0.073^a^  p=0.649 | r=0.175  p=0.215 | r=0.054^a^  p=0.737 | r=0.091  p=0.520 | r=-0.098^a^  p=0.541 | r=0.154  p=0.276  q=0.325 | r=-0.090^a^  p=0.575  q=0.610 | r=0.080  p=0.572 |
| **Psychological well-being** | r=-0.083^a^  p=0.606 | r=0.038^a^  p=0.796 | r=-0.069^a^  p=0.669  q=0.765 | r=0.060^a^  p=0.680 | r=-0.062^a^  p=0.700 | r=0.034^a^  p=0.814 | r=-0.040^a^  p=0.805 | r=0.016^a^  p=0.914 | r=-0.044^a^  p=0.787 | r=-0.051^a^  p=0.723 | r=-0.117^a^  p=0.464 | r=0.173^a^  p=0.230  q=0.325 | r=-0.127^a^  p=0.429  q=0.572 | r=0.005^a^  p=0.971 |
| **Symptoms’ influence on daily life** | r=-0.090^a^  p=0.577 | r=0.169  p=0.231 | r=-0.173  p=0.279  q=0.656 | r=0.093  p=0.511 | r=-0.127^a^  p=0.429 | r=0.012  p=0.930 | r=-0.165^a^  p=0.302 | r=0.178  p=0.206 | r=-0.110^a^  p=0.492 | r=0.203  p=0.148 | r=-0.056^a^  p=0.726 | r=0.254  p=0.069  q=0.235 | r=-0.260^a^  p=0.100  q=0.267 | r=0.100  p=0.480 |
| **Total IBS-SSS** | r=-0.065^c^  p=0.699 | r=0.136  p=0.335 | r=-0.334^c^  p=0.041  q=0.328 | r=0.004  p=0.978 | r=-0.221^c^  p=0.183 | r=-0.139  p=0.326 | r=-0.281^c^  p=0.087 | r=-0.108  p=0.446 | r=-0.076^c^  p=0.651 | r=-0.021  p=0.881 | r=0.117^c^  p=0.484 | r=0.304  p=0.029  q=0.232 | r=-0.376^c^  p=0.020  q=0.160 | r=-0.021  p=0.885 |

Correlation analyses were performed in 43 patients with POTS and 52 healthy controls using Spearman’s correlation analysis. Q = P-values adjusted for false discovery rate according to the Benjamini-Hochberg procedure. A q-value <0.05 was considered statistically significant. GIP: glucose-dependent insulinotropic peptide; GLP-1: glucagon-like peptide-1; PYY: peptide YY; IBS-SSS: irritable bowel syndrome severity scoring system.

^a^2 missing

^b^1 missing

^c^5 missing
